# Transesophageal echocardiography findings of post-deployment transcatheter aortic valve replacement complications and valve-in-valve approach

**DOI:** 10.1186/s44348-024-00018-1

**Published:** 2024-06-25

**Authors:** Otito Ojukwu, Kishore Balasubramanian, Stuart Lander, Robert Hebeler, Saravanan Ramamoorthy

**Affiliations:** 1 Department of Anesthesia and Critical Care, Texas A and M School of Medicine, College Station, TX USA; 2 Department of General Surgery, Texas A&M School of Medicine, College Station, TX USA; 3 Baylor Scott and White Heart and Vascular Hospital, Baylor University Medical Center, Dallas, TX USA; 4 Department of Anesthesiology and Perioperative Medicine, US Anesthesia Partners, Dallas, TX USA; 5 Department of Anesthesiology, Baylor University Medical Center, Dallas, TX USA; 6 Department of Anesthesiology, Texas A&M School of Medicine, College Station, TX USA

**Keywords:** Transaortic valve replacement, Echocardiography, Cardiothoracic surgery

A 73-year-old man with a past medical history of moderate coronary arterial disease, hypertension, hyperlipidemia, obstructive sleep apnea, and obesity with a body mass index of 39 kg/m^2^ presented with severe symptomatic aortic stenosis requiring a transcatheter aortic valve replacement (TAVR) procedure. Patient had moderate calcifications of the aortic and tricuspid valve with an aortic valve calcium score of 489 Agatston units on noncontrast computed tomography imaging. The peak aortic valve velocity was 4.3 m/sec, with a mean gradient of 47 mmHg, and an estimated valve area of 0.7 cm^2^. He underwent transesophageal echocardiography (TEE) and general anesthesia due to severe obesity and inadequate transthoracic echocardiography (TTE) window.

The TAVR procedure was performed using a transfemoral access approach. The valve was then fully deployed, and the delivery sheath was recaptured and removed. At the end of deployment, positioning of the valve through its course was verified using TEE, which noted a dislodged 29-mm Evolut (Medtronic) in its transition to the ascending aorta. A 6F snare was utilized to retrieve the valve and it was pulled towards the arch; however, it would not stay, nor would the valve deflect enough to be pulled into the descending aorta. Due to the difficulty in accessing the Evolut valve the team elected to proceed with a 26-mm Edwards Sapien 3 (Edwards Lifesciences Corp).

A unique valve-in-valve approach was then utilized to replace the dislodged Evolut FX valve (Medtronic). The Sapien valve was prepared and the DrySeal (Gore Medical) was exchanged for a 22 E-sheath. Using the same right femoral artery that was previously accessed, the Sapien was deployed through the previously lodged Evolut and situated. TEE verified good position of the Sapien 3 as well as the displaced Evolut valve (Fig. 1A, B). After proper placement of Sapien, the Evolut valve was positioned at the arch and was still difficult to retrieve. The decision was made to convert to cardiopulmonary bypass (CPB) and aortotomy to remove the Evolut FX valve.

**Fig. 1:**
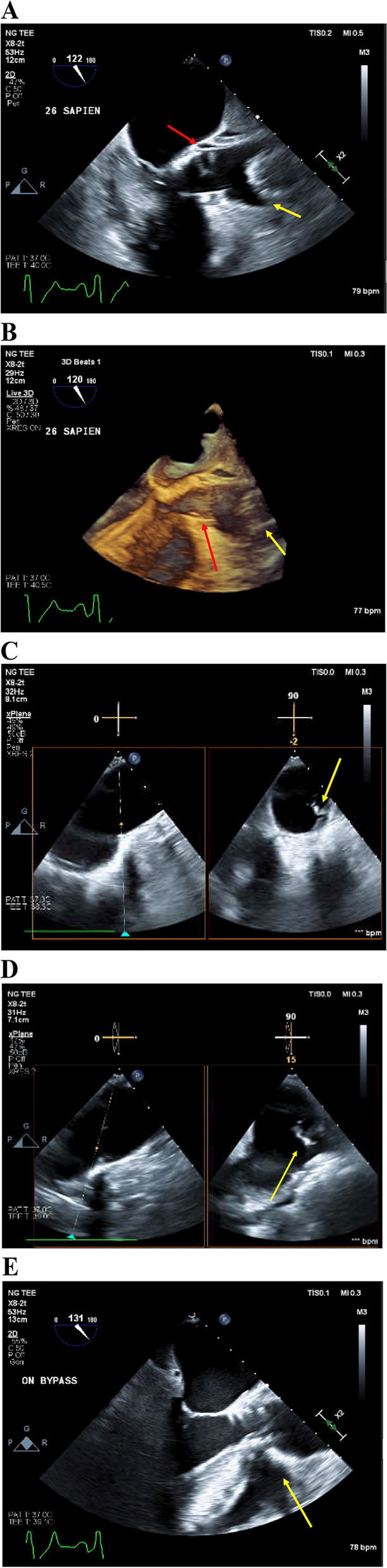
**A** Transesophageal echocardiography (TEE) mid esophageal long axis 122° 2-dimensional (2D) image showing proper placement of the 26-mm Sapien valve (red arrow) and proximal portion of displaced 29-mm Evolut valve (yellow arrow). The aortic tear located at distal portion of the Evolut valve could not be visualized because the dislodged valve was obstructing the view. **B** TEE mid esophageal long axis 120 3D imaging showing proper placement of the 26-mm Sapien valve (red arrow) and proximal portion of displaced 29-mm Evolut valve (yellow arrow). **C**, **D** TEE short and long axis view without color flow doppler of the aorta showing a 5-mm aortic intimal tear. **E** TEE mid esophageal at 131° 2D image, showing the 26-mm Sapien valve in proper placement

After the Evolut valve was removed from the aorta, TEE revealed a 5-mm aortic tear (Fig. 1C, D). This was not appreciated previously because the dislodged valve was blocking the view of the aortic tear. The tear was immediately apparent, spanning the anterior one-third of the aorta with tears in several other sections. The flap was repaired using two felt strips, which were then reinforced with a prolene double layer and glue.

Figure 1E shows the final position of the Sapien valve after removal of the Evolut. The pigtail was reinserted, and hemodynamics were checked and determined to be satisfactory. TEE was used to assess final positioning and perivalvular leaks which were satisfactory. The heart came back to sinus rhythm and the patient was weaned off CPB. Sternotomy wound was closed, and the patient was taken to the intensive care unit in stable condition. Patient was discharged from the intensive care unit on postoperative day 3.

In terms of intraoperative imaging in TAVR procedures, the primary decision is between TEE and TTE. TEE provides high quality imaging of cardiac structures. It requires the insertion of a probe into the esophagus to obtain cardiac valvular images with limited interreference from patients’ body habitus [1]. Additionally, it allows for rapid and early detection of intraoperative complications in comparison to TTE [2]. TTE, on the other hand, is noninvasive, widely accessible, and can be used on-demand. However, TTE images are commonly obscured based on patients’ body habitus, chest wall, tissue, or lung hyperinflation [2].

The benefit of using TEE was seen at multiple points during this case. Despite multiple attempts of Evolut valve placement, the valve still dislodged. TEE allowed for real time identification of the dislodgment and early identification of the subsequent aortic dissection. Other imaging modalities may not have detected the dissection at the early stages. Real time visualization also provided the information necessary to convert to a rapid CPB and facilitated the decision for a valve through valve placement [3]. If another imaging procedure had been used, significant time would have been taken to convert to TEE imaging and general anesthesia.

### Supplementary Information

Additional file 1: Transesophageal echocardiography 123° 2-dimensional image showing displaced 29-mm Evolut valve

Additional file 2: Transesophageal echocardiography mid esophageal long axis 122 2-dimensional image, showing proper placement of the 26-mm Sapien valve and proximal portion of displaced 29-mm Evolut valve.

Additional file 3: Transesophageal echocardiography mid esophageal long axis 120 3-dimensional imaging showing proper placement of the 26-mm Sapien valve and proximal portion of displaced 29-mm Evolut valve.

Additional file 4: Transesophageal echocardiography short and long axis view without color flow doppler of the aorta showing a 5-mm aortic intimal tear.

Additional file 5: Transesophageal echocardiography mid esophageal long axis 131 2-dimensional image**,** showing the 26-mm Sapien valve in proper placement.

## Data Availability

Not applicable.

## Abbreviations

CPB: Cardiopulmonary bypass
TAVR: Transcatheter aortic valve replacement
TEE: Transesophageal echocardiography
TTE: Transthoracic echocardiography
